# Fermented Foods and Food Microorganisms: Antioxidant Benefits and Biotechnological Advancements

**DOI:** 10.3390/antiox13091120

**Published:** 2024-09-16

**Authors:** Myung-Ji Seo

**Affiliations:** 1 Division of Bioengineering, Incheon National University, Incheon 22012, Republic of Korea; mjseo@inu.ac.kr; 2 Department of Bioengineering and Nano-Bioengineering, Incheon National University, Incheon 22012, Republic of Korea; 3 Research Center for Bio Materials & Process Development, Incheon National University, Incheon 22012, Republic of Korea

Fermented foods have been a part of human civilization since ancient times, offering enhanced flavors, extended shelf-life, and improved nutritional value through the action of microorganisms [1]. The fermentation process has been pivotal in transforming various food substrates, ranging from dairy and vegetables to cereals and legumes, into staples of diets around the globe [2,3,4]. One of the most compelling health benefits of fermentation lies in its ability to significantly boost the antioxidant capacity of foods [5,6].

The rising demand for plant-based protein and non-dairy milk alternatives has highlighted soybeans and soymilk for their nutritional benefits. Fermentation using lactic acid bacteria (LAB) like *Lacticaseibacillus paracasei* modifies the nutritional profile of soy-derived products and provides a rich source of antioxidant peptides, which are small enough to be absorbed and utilized by the body [7]. Peptides from *L. paracasei* MK1-fermented soymilk exhibited antioxidant activity through mechanisms involving the inhibition of reactive oxygen species (ROS), which are known to exacerbate skin aging by inducing oxidative damage to skin cells. Lee et al. [8] demonstrated that certain synthesized peptides contributed to anti-aging effects, enhancing procollagen production and reducing pro-inflammatory cytokine levels in UVB-damaged skin cells, thus mitigating the negative effects of photoaging by reducing oxidative stress, promoting skin elasticity, and reducing wrinkle formation. Letizia et al. [9] fermented soymilk using *Lactiplantibacillus plantarum* LP95 and demonstrated a significant enhancement of the antioxidant properties of the soymilk. As a strain with notable β-glucosidase activity, *L. plantarum* LP95 efficiently converted isoflavone glucosides such as genistein, glycitein, and daidzein into their more bioavailable aglycones during fermentation. The process improved the organoleptic properties and shelf-life stability, and increased isoflavone concentrations and antioxidant capacity, indicating the ability of the fermented product to mitigate oxidative damage in numerous health conditions.

Fermentation offers a biotechnological approach to enhance the bioaccessibility of antioxidant compounds in plants, freeing them from glycosides and plant matrix complexes. In almonds, microbiota modulation varied between cultivars, and the Guara cultivar supported a distinct gut microbial community structure, promoting beneficial genera like *Dorea*, which is associated with dietary fiber degradation. Guara exhibited the highest total antioxidant capacity and the highest concentration of short-chain fatty acids (SCFAs), promoting gut health by serving as energy sources for intestinal cells and modulating the immune response [10]. In herbal infusions, lacto-fermentation by *L. plantarum* enhanced the phenolic content of infusions with thyme, rosemary, echinacea, and pomegranate peel, and led to modulated anti-inflammatory effects and an increased antioxidant capacity [11].

The use of fermentation has also been explored for the transformation of by-products and waste materials to obtain new products with improved quality [12]. The use of microbial inoculants in fermentation has further improved the taste, texture, and nutritional profile of foods. Functional fruit wines combine the flavors of fruit with added health benefits, and upcycled by-products are valuable alternatives for nutraceutical development and reducing environmental waste. The health benefit potential of grape pomace, a by-product of the wine industry, was confirmed through fermentation with LAB which enhanced its functional properties for use in sourdough bread [13]. The fiber- and polyphenol-rich grape pomace, typically discarded or used for animal feed, was able to be used as a functional food ingredient. Fermented pomace exhibited significantly higher antioxidant activity and down-regulation of pro-inflammatory cytokines, in addition to the enhanced flavor profiles of the produced bread. Likewise, Li et al. [14] used various parts of green bananas, including the peel, pulp, and whole fruit, to develop functional fruit wines. Overall, the wines showed higher antioxidant activities than their raw fruit counterparts, and the peels, typically considered waste, maintained the highest levels of specific phenolics, tannins, and flavonoids. Peel wine had the greatest potency against oxidative stress induced by hydrogen peroxide in cell-cultured based assays, along with a better capacity to scavenge superoxide anions, highlighting their potential as a rich source of antioxidants with anti-inflammatory and cardioprotective effects.

The aromatic profile of Catarratto wines, a non-aromatic white grape variety from Sicily, was enhanced by using *Metschnikowia pulcherrima* in combination with the sequential inoculation of *Saccharomyces cerevisiae* and the addition of a glutathione-rich inactivated yeast [15]. The inactivated yeast helped limit oxidation, thus preserving the color and stability of the aromatic compounds, particularly by protecting esters and volatile thiols from oxidative degradation. The resulting wines had an enhanced aromatic complexity, fruity and floral aromas, and improved oxidative stability and preservation. The addition of GIY protected the wine from oxidative damage and allowed for the preservation of key aromatic compounds, while *M. pulcherrima* significantly contributed to the production of desirable esters. As an alternative to wine, kombucha is a popular drink with various health benefits and a growing variety of substrates used in its production. Xiong et al. [16] developed kombucha beverages using bamboo leaf and mulberry leaf as alternative substrates for fermentation. These caffeine-free alternatives to traditional kombucha possess strong antioxidant activities and various bioactive compounds.

Fermentation not only enhances the concentration of antioxidants but also modifies their molecular structure, increasing their solubility and bioavailability, making them more effective in human bodies [17]. In individuals with metabolic conditions such as hyperglycemia and hyperlipidemia, fermentation plays a key role in elevating the bioavailability of antioxidants for overall metabolic health and the management of non-communicable diseases like diabetes and cardiovascular disease [18]. Black goji berry juice fermented with *Lactobacillus rhamnosus* significantly enhanced its sensory qualities and functional properties, particularly in inhibiting the key steps involved in lipid digestion and absorption [19]. Fermentation converted complex polyphenols into simpler, more bioactive compounds and increased lactic acid production and probiotic viability. The fermented product showed enhanced abilities to bind bile acids, inhibit cholesterol micelle formation, and increase pancreatic lipase inhibition, all crucial activities in reducing cholesterol absorption and lipid digestion. The fermented berries also demonstrated improved inhibition of an enzyme that degrades incretin hormones crucial for glucose regulation. This inhibition extends the activity of incretins like GLP-1 and GIP, enhancing insulin secretion, reducing blood sugar levels, and allowing for the potential dietary intervention of managing hyperglycemia in diabetic individuals. Wang et al. [20] presented the role of quorum-sensing transcription factor SdiA in regulating NADPH metabolism and (*S*)-equol production in the probiotic *Escherichia coli* Nissle 1917. (*S*)-Equol, a metabolite derived from dietary isoflavones like daidzein, has significant antioxidative and estrogenic activities. The researchers identified a bottleneck in (*S*)-equol production linked to NADPH availability and provided insights into optimizing engineered probiotics for (*S*)-equol production by manipulating quorum-sensing pathways and improving NADPH regeneration to enhance the health benefits of dietary isoflavones, especially in individuals who cannot naturally produce (*S*)-equol.

Fermentation has also been exploited for the benefit of livestock, aiding in their general health and also in reducing the need for antibiotics. The study on *Limosilactobacillus reuteri* PSC102 highlights its significant probiotic, antioxidant, and antibacterial properties, suggesting its potential as a functional feed additive, particularly for swine [21]. A standout feature of this strain is its strong antioxidant activity, as evidenced by its ability to scavenge free radicals, and through its reducing power. These antioxidant activities are vital for mitigating oxidative stress-related disorders which are a common challenge in farm animals, especially during periods of rapid growth or infection. The antioxidant properties of the microorganism suggest that it could enhance the health and growth performance of animals by counteracting the damaging effects of ROS. Qiu et al. [22] demonstrated that yeast culture supplementation in laying hens had significant benefits on antioxidant capacity, egg production performance, egg quality, intestinal morphology, and microbial composition. These improvements were attributed to enhanced nutrient absorption and metabolism, facilitated by the beneficial components of the cultures, such as amino acids, peptides, and vitamins, which optimize digestive and metabolic functions, and the oligosaccharides and other bioactive compounds that enhance antioxidant enzyme activity and neutralize free radicals. Yeast culture supplementation increased serum total antioxidant capacity and glutathione peroxidase activity, while reducing malondialdehyde levels, which are markers of oxidative stress. The improvement in antioxidant activity highlights the potential of the cultures to mitigate oxidative damage in laying hens, thereby supporting overall health and longevity. Zhao et al. [23] delved into the role of *Lactobacillus* in reducing oxidative stress, with a focus on its potential in preventing and treating diseases such as inflammatory bowel disease (IBD), cancer, and liver-related diseases. The antioxidant properties of *Lactobacillus* have been widely studied in cell lines, animal models, and some clinical reports, showing promising results in mitigating oxidative damage and inflammation. The *Lactobacillus* genus commonly found in the gastrointestinal tract can produce antimicrobial metabolites and modulate the host immune system, as some strains possess oxidative stress resistance genes, while others produce exopolysaccharides (EPS) that protect cells from oxidative damage. These microorganisms have shown potential in increasing the host’s antioxidant capacities by enhancing enzyme activity or directly neutralizing ROS, and studies have shown that *Lactobacillus* strains can positively influence the redox balance and mitigate oxidative stress. Siziya et al. [24] explored the antioxidant potential of C_30_ carotenoids produced by microorganisms, highlighting their significance and advantages in various industries such as food, feed, cosmetics, and pharmaceuticals. While carotenoids are known for their antioxidant, anti-inflammatory, and anticancer properties, which protect cells from oxidative damage, C_40_ carotenoids dominate both research foci and commercial markets compared to their less abundant C_30_ counterparts [25,26]. The paper discusses the ROS quenching capabilities and oxidative stress reduction potential of the C_30_ carotenoids, which may aid in preventing cellular damage. The study discusses the metabolic pathways of microbial carotenoids and the potential for large-scale, eco-friendly production through biotechnological advancements. Nemes et al. [27] focused on integrated technologies for cereal bran valorization, aiming to improve the bioaccessibility and bioavailability of bioactive components such as phenolic acids, dietary fibers, and biopeptides that are naturally bound within the bran matrix. These bioactive compounds have demonstrated potential health benefits, including anti-inflammatory, antioxidant, anticancer, and cardiometabolic protective effects, which tend to be limited due to their insoluble and bound forms in the bran. An integrated approach was proposed, involving pretreatment methods such as physical, chemical, and enzymatic techniques, followed by microbial fermentation to release these bound components for enhanced utilization in functional foods and supplements. Solid-state and submerged fermentation improve the release of bioactive compounds from bran with microorganisms such as *Aspergillus*, *Lactobacillus*, and *Saccharomyces*, which can break down cell wall components, releasing phenolics, peptides, and other bioactive compounds. Biopeptides were noted as having antihypertensive, antioxidant, and antimicrobial properties, with oats and rice bran being particularly rich sources of bioactive peptides. Phenolic compounds in bran scavenge free radicals, reducing oxidative stress and potentially lowering the risk of chronic diseases while bran components like ferulic acid and biopeptides have been linked to reduced inflammation in clinical studies.

The papers of this Special Issue provide a glimpse into the current state of research into fermented foods and the roles of food microorganisms in the specific aspect of antioxidant activity. As the demand for natural, health-promoting foods continues to rise, fermented products represent a fusion of ancient culinary practices and cutting-edge science, offering sustainable solutions to improve public health and combat diseases driven by oxidative stress. The growing body of research in this field underscores the transformative potential of fermentation biotechnology, not only for improving food preservation and safety but also for delivering potent therapeutic benefits.
